# Photoluminescent Sensor of Scarification Efficiency of Fodder Plants’ Seeds

**DOI:** 10.3390/s23010106

**Published:** 2022-12-22

**Authors:** Mikhail V. Belyakov

**Affiliations:** Federal Scientific Agroengineering Center VIM, 109428 Moscow, Russia; bmw20100@mail.ru; Tel.: +7-991-688-9404

**Keywords:** forage plants, seeds, scarification, germination, photoluminescence, linear regression model

## Abstract

Optoelectronic sensors open up new possibilities for predicting the yield for their possible correction, including increasing the seed germination of forage plants. The luminescent properties of unscarified and scarified seeds of various germination galega, clover and alfalfa are compared. The dependence of germination on the photoluminescence flux is approximated by linear equations with a determination coefficient *R*^2^ = 0.932–0.999. A technological process for analyzing the scarification quality of forage seed plants is proposed, including sample preparation, photoluminescence excitation and registration, amplification of the received electrical signal and determination of germination based on calibration equations. This is followed by a decision on sowing, or re-scarification. The scheme of the scarification quality control device has been developed for which the LED, as well as the radiation receiver and other elements, has been selected according to the energy efficiency criterion. Mechanical scarification of the forage plants’ seed surfaces has a significant effect on their photoluminescent properties. The flux increases by 1.5–1.7 times for galega, 2.0–3.0 times for clover and 2.3–3.9 times for alfalfa. Linear approximation of the flux dependence on germination with a high coefficient of determination allows us to obtain reliable linear calibration equations. Preliminary mock-up laboratory tests allow us to talk about the developed method’s effectiveness and device.

## 1. Introduction

The application of rapidly developing digital technologies, Internet of Things (IoT) systems, robotic complexes and artificial intelligence will allow the greatly increased efficiency of agricultural industry, including animal husbandry. It is possible to increase labor productivity, to reduce energy and material costs and to ensure the ecological safety of the agricultural industry and the environment. Developing the concept of intelligent agriculture, the following are areas of digital technology application as integrated management of agricultural industry and digital technologies in crop production, animal husbandry, energy supply, storage and processing of agricultural products.

It is necessary to have a fodder base for the sustainable development of dairy and beef cattle breeding, one of the important components of which is the cultivation of forage plants (galega, clover, etc.). These plants have seeds with hard shells. Scarification must be carried out, therefore, to increase their germination. For this purpose, scarifiers have been created [1].

Such optical methods as RGB imaging, multi- and hyperspectral sensors, thermography or chlorophyll fluorescence have proven their potential in automated, objective and reproducible detection systems for identifying and quantifying plants at all stages of their development. When used on various platforms (portable, ground-based, airborne, etc.), these optoelectronic sensors open up new opportunities for predicting crop yield and plant quality for their possible correction.

The purpose of this study is to develop an optical photoluminescent diagnostics device for the scarification effectiveness of forage plant seeds.

Remote sensors are increasingly being used to monitor plant conditions. They provide nondestructive spatial detection and quantification of plants at various measurement levels [2]. 

In recent years, there has appeared a new generation of devices based on miniature spectrometers. They operate in the near-infrared (NIR) range of the spectrum as a rule [3]. Often the NIR spectroscopy method is used for routine analysis of samples from forage producers, selectionists, animal nutrition specialists, livestock breeders and forage production companies. The study [4] compared the prediction efficiency of three different portable devices by comparison to a desktop laboratory NIR device that uses a wide range of pretreated dried forage samples of alfalfa and grass. Near-infrared spectroscopy with a console for determining the reflection coefficient was used for direct analysis of samples when sorting weed rice from cultivated rice [5]. 

Portable devices have been developed for measuring the reflection coefficient in the near-infrared region for evaluating forage on farms [6], including the use of IoT [7], for predicting crude protein and acid detergent fiber, etc. [8]. A portable near-infrared spectroscopy system was developed for the rapid measurement of the water content in rapeseed leaves in the spectrum range from 900 nm to 1700 nm [9] and monitoring the quality of sugar cane [10,11].

A crop chlorophyll detector based on an optical sensor with an interference filter was proposed for the nondestructive determination of chlorophyll content in field crops based on the characteristics of chlorophyll reflection in the visible and near-infrared spectrum 400–1000 nm [12]. A combined spectrometer in the near infrared region has been tested to measure protein concentration in the spectral reflection range from 850 nm to 1040 nm [13].

The study [14] presents a portable sensor detector of the sweetness and hardness degree of kiwi. It consists of a control/processing unit, an LED panel and a driver unit, a light signal detection and amplification unit, an input/output unit and a battery. It uses a microcontroller STM32 to collect and process data. LEDs of 1000 nm and 1100 nm are used as a light source. The reflected light from the kiwi is recorded by a silicon pin photodiode. A portable spectrometer with a photodiode array Vis/NIR operating in reflection mode is used to measure the contents of soluble solids in melon fruits [15] and to determine the ripeness of grape fruits [16,17]. 

To measure the degree of olive maturation, a diagnostic tool calibrated using image analysis operating in the visible and near infrared range is proposed. It receives an RGB image. Spectroscopic analyses are performed using a desktop FT-NIR and a portable Vis/NIR instrument. The desktop device is equipped with a fiberoptic probe, and it recorded the spectra in the range from 800 nm to 2500 nm [18]. The same method is used by the olive-oil diagnostics [19]. A laboratory sample of a device for determining stress conditions of plants was developed and tested on plants of the garden basil variety [20].

## 2. Materials and Methods

The luminescent properties of scarified and scarified galega, clover and alfalfa seeds of various germinations were studied. Mechanical scarification of 400 seeds of all the studied forage plants was carried out using sandpaper (to simulate the operation of an industrial scarifier). The spectral characteristics of excitation and luminescence before and after seeds scarification were measured with control of germination of the initial and scarified samples. First scarification was performed once and repeatedly after spectral measurement for the same sample.

Measurements of the excitation (absorption) spectra η_e_(λ) and photoluminescence (emission) spectra φ*_l_*(λ) were carried out on a diffraction spectrofluorimeter, the Fluorat-02-Panorama (Lumex, Russia), according to a previously approved method described in [21]. The spectrofluorimeter consists of an optical system with a light source (a pulsed xenon lamp) and a light receiver (a photoelectronic multiplier). The lamp operates in the mode of short (1.5 microseconds) pulses with a repetition rate of 25 Hz. To isolate the required spectral range, it uses monochromators with concave diffraction gratings operating in the first order of diffraction.

For the mathematical processing, the integrals under the luminescence curves were determined in special built-in software, PanoramaPro, and their average values were found for 100 measurements.

(1) $$\Phi =\overset{\lambda_{2}}{\underset{\lambda_{1}}{\int }}\varphi_{l}\left(\lambda \right)d\lambda$$

where φ*_l_*(λ) is the spectral characteristics of photoluminescence, and λ_1_, …, λ_2_ are the limits of the operating spectral range of photoluminescence.

The values of the photoluminescence flux Φ are calculated in relative units (r.u.) and converted into lumens (lm) by calibration according to a reference source and a luxmeter.

## 3. Results and Discussion

It was previously found that the excitation spectra η_e_(λ) of galega seeds are located in the spectral range of about 400–500 nm and have two peaks at 462 nm and 485 nm [1]. The photoluminescence spectra φ*_l_*(λ) are single modal with a peak at about 525–535 nm and are located in the spectral range of 480–620 nm. The spectra shift upward after scarification [22]. The situation is similar for tetraploid clover [23] and alfalfa [1].

**Figure 1:**
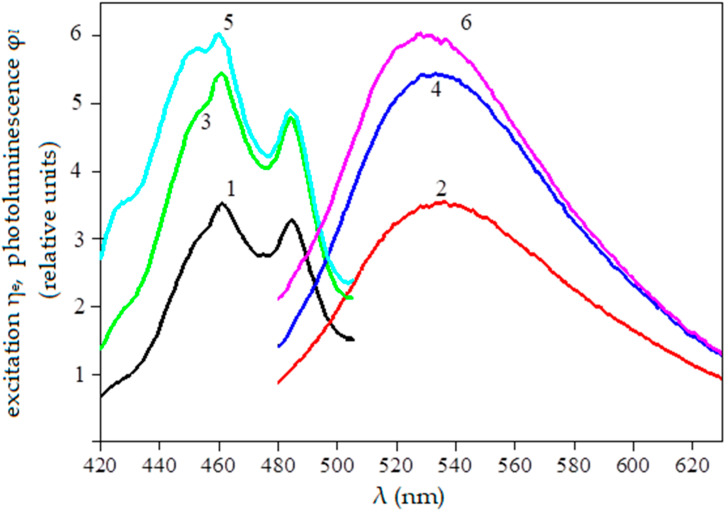
The spectral characteristics of unscarified and scarified galega seeds: 1,2—uncarified, 3,4—scarified once, 5,6—scarified twice.

The luminescence spectra of double-scarified seeds were used to determine the dependence of photoluminescence flux on seed germination after scarification. Double scarification was chosen based on the scarifiers’ manufacturability because carrying out multiple scarifications in production conditions is economically unprofitable. With each repeated scarification, the proportion of destroyed seeds increases and energy and time are required. Therefore, in practical crop production, as a rule, one scarification is used—less often two. The spectral characteristics of unscarified and scarified galega seeds (for example) are shown in Figure 1.

Data of seeds germination before and after scarification were used to assess the quality of scarification (Table 1).

**Figure 2:**
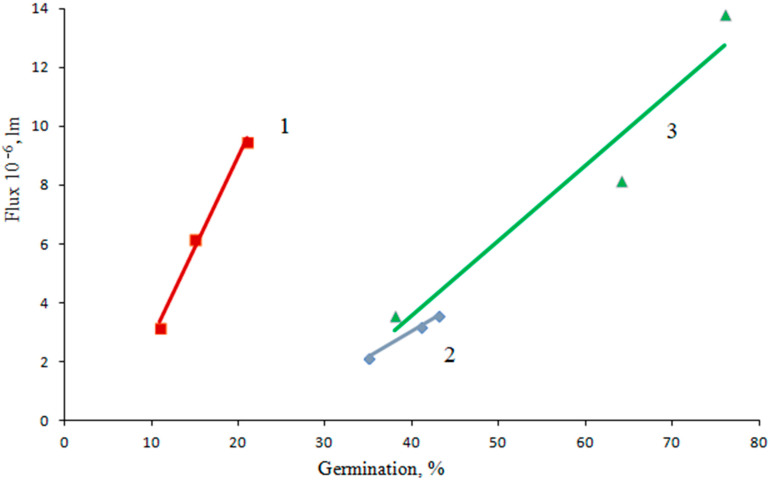
Approximation of the dependence of photoluminescence flux on the germination of forage plant seeds with double scarification: 1—clover, 2—galega, 3—alfalfa.

For the seeds of the studied forage plants, the dependence of the germination value on the photoluminescence flux is shown in Figure 2.

It is convenient to use the inverse dependence *B*(Φ) for the practical implementation of the method. A linear approximation was carried out and the following equation was obtained for the galega seeds:

(2) $$B=5.62\cdot \Phi \cdot 10^{6}+22.9$$

where *B* is the germination value (%), and Φ is the photoluminescence flux (lm).

The determination coefficient is *R*^2^ = 0.9999, which indicates a high extent of accuracy and reliability of the approximation.

The linear approximation equation of clover seeds is described by the equation:

(3) $$B=1.59\cdot \Phi \cdot 10^{6}+5.72$$

The determination coefficient is *R*^2^ = 0.9919. This extent of accuracy and reliability of the approximation is sufficient.

The linear approximation of alfalfa seeds is described by the equation:

(4) $$B=3.66\cdot \Phi \cdot 10^{6}+28.2$$

The determination coefficient is *R*^2^ = 0.9315.

It is possible to use the same light source to analyze the studied plant seeds because the peaks of the excitation spectra of galega (460 nm), clover (448 nm) and alfalfa (450 nm) are located very close. The same photoluminescence detection range for these cultures can also be used, since the peaks of the luminescence emission spectra are also located close to each other (516 nm for clover, 517 nm for alfalfa and 536 nm for galega).

**Figure 3:**
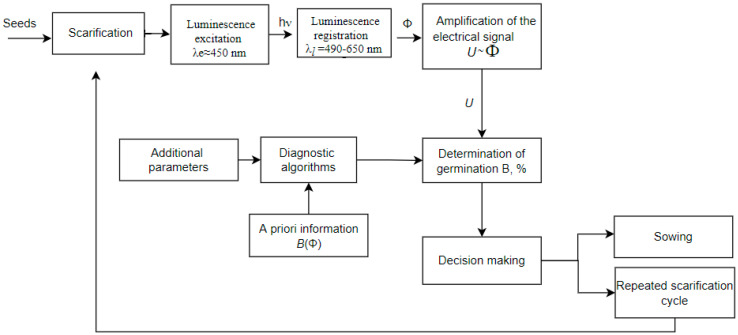
Block diagram of the technological process of express analyzing the scarification quality of forage plant seeds.

The technological process of analyzing the scarification quality of forage plant seeds is proposed, the block diagram of which is shown in Figure 3.

1. Forage plants seeds such as galega, clover and alfalfa undergo a process of mechanical scarification. After it, all of the scarified seeds or an experimental sample enter a dark light-tight housing. It is also possible to integrate the technological process of rapid germination diagnostics into the technological processes of scarification for continuous monitoring of its effectiveness.
2. Simultaneously with the first stage, the type of seeds (culture, sport) and other seed parameters, such as humidity or clogging, can be measured (or can be set otherwise, for example, according to the supporting documentation), which is necessary to establish the appropriate diagnostic algorithm.
3. The photoluminescence of seeds is excited by light of a narrow spectral range with a peak λ_e_ ≈ 450 nm (448–460 nm) within 20 μs.
4. A signal proportional to the photoluminescence flux Φ in the spectral range from 490 nm to 650 nm is recorded. The process takes 2–3 s with the results averaged.
5. The received proportional to the photoluminescence flux Φ photosignal (photovoltage *U*, photocurrent *I*) is amplified by an amplifier.
6. The amplified photo signal enters into the microprocessor. There the signal is processed, taking into account the a priori information available in its memory. This is the linear characteristic *B*(Φ) obtained for scarified seeds and selected according to the diagnostic algorithm for a specific culture.
7. A decision on further actions with seeds is made based on the results of the germination determination. These can be sowing (with sufficient germination value) or repeated scarification to increase germination before sowing with repeated express control.

It is advisable to take measurements several times with the averaging of the obtained results or continuously in the seed stream.

**Figure 4:**
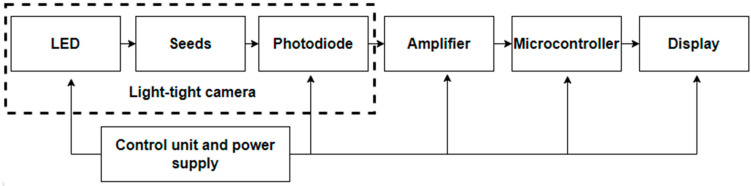
Block diagram of the seed scarification quality-control device.

**Figure 5:**
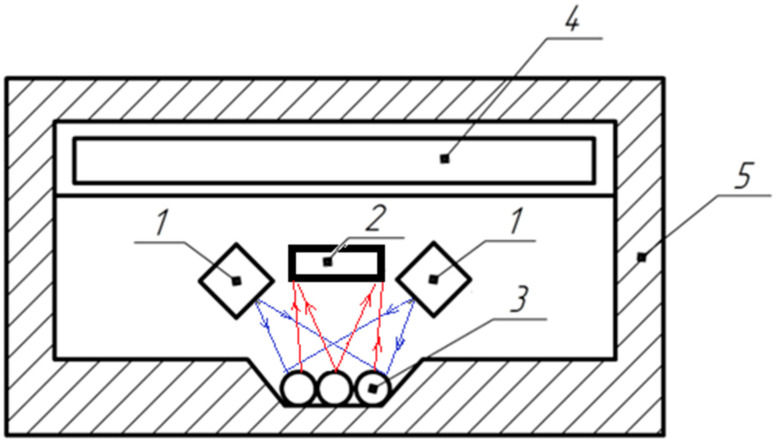
Functional diagram of the device: 1—light source; 2—light receiver; 3—tested seeds; 4—electronic unit; 5—light-tight housing.

Structural (Figure 4) and functional (Figure 5) schemes of the device that implement developed technology are proposed.

LEDs were chosen as light sources to excite photoluminescence. The main criterion for choosing light sources is the accordance of its spectrum to the sensitivity spectrum of seeds (luminescence excitation spectrum η_e_(λ)). In this case, the maximum efficiency of the source is obtained, especially if the LED spectrum maximum is superimposed on the maximum of the seed excitation spectrum curve. However, while developing the device, it is necessary to take into account that the ranges of the source light spectrum and the receiver sensitivity spectrum do not overlap. Otherwise, it is worth using cutting-off light filters or temporary separation switching on the source and receiver.

The quantitative criterion for choosing an LED is its effective output by excitation. The effective light output for the chosen LED is calculated by the formula:

(5) $$k_{e,e}=\frac{\Phi_{eff}}{\Phi_{full}}=\frac{\int_{0}^{\infty }\varphi {\left(\lambda \right)}_{\mathrm{LED}}S\left(\lambda \right)d\lambda }{\int_{0}^{\infty }\varphi {\left(\lambda \right)}_{\mathrm{LED}}d\lambda },$$

where Φ*_eff_* is the effective flux; Φ*_full_* is the full flux of exciting light; *S*(λ) is the spectral sensitivity of seeds; and φ(λ)_LED_ is the LED light spectrum. 

The calculation results are shown in Table 2.

After analyzing the results of calculating the effective return of light, it can be argued that LED 150353BS74500 is the most advantageous to use as a light source, since its effective light output is the highest. 

A photodiode *BPW*21*R* from the *Vishay* company was chosen as a light receiver [29].

It is necessary to use a measurement delay for preventing the overlap of the ranges of the light source and the sensitivity of the receiver in the short-wavelength region of the spectrum. The time between the lighting of the seeds and the measurement of the photoluminescence flux should be 0.75 ms.

The *AD*820*ANZ* was chosen as an operational amplifier [30]. The amplifier *AD*820*ANZ* is a precision, low-consumption operational amplifier with an input stage on field-effect transistors. The *ATmega328P* was chosen as a microcontroller [31]. The *ATmega328P* is the AVR group microcontroller. It has an 8-bit processor and allows the execution of the majority of commands in one clock cycle.

A liquid crystal display, *LCD*1602, was chosen for visualization [32]. This display is based on liquid crystal technology. Two factors should be taken into account when designing electronic devices. The first is that an inexpensive device is needed to display information. The second equally important factor is the availability of prepared libraries for *Arduino*. The most commonly used of all the available LCD displays on the market is the *LCD* 1602. It can show *ASCII* symbols in two lines (16 symbols in one line), where each symbol is a 5 × 7 pixel matrix.

The main elements of the sensor being developed are presented in Table 3.

The developed electrical circuit diagram includes two independent functional cascades. The total voltage of the electrical circuit is unipolar by +5 V. The power supply is carried out using a 9 V Crohn battery and stabilizer, which lowers voltage to 5 V. Switching the device on is organized by a key. The voltage indication in the device circuit is carried out using an LED.

The first functional unit consists of two-position analog switches. It serves for the organization of a pulsed power supply for the LEDs and light-emitting diodes included in the classical scheme with current-limiting resistors. The time between pulses is 0.75 ms and is set by the microcontroller. LEDs are purposed to excite the seed surfaces with a light source of the required range.

The purpose of the second cascade is to receive and process the photoluminescence flux of the forage plant seed surfaces. It consists of a photodiode and an operational amplifier. The photodiode operates in the valve mode. The processed information is sent to the visualization device.

The case should consist of several parts for the purpose of convenient installation and quick repair during further operation of the device. It includes the base of the housing, the device cover, mounts for LEDs and photodiodes, a mount for components, a measuring chamber, a measuring chamber cover and handles for mounting on the measuring chamber cover. These parts were made of durable polyamide plastic by casting. It is also possible to use a *3D* printer to make them. The overall dimensions of the device layout are 225*110*195 mm, with weight of about 2 kg. This is much less than the mass and size indicators of a universal stationary spectrofluorimeter.

**Figure 6:**
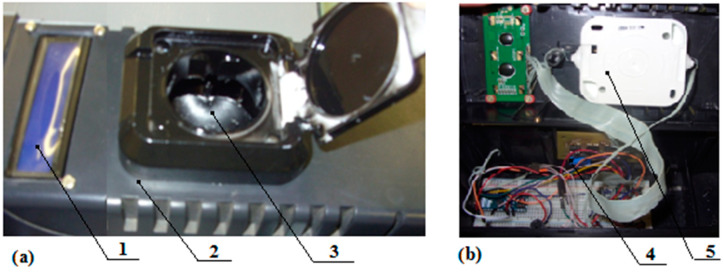
Exterior (**a**) and interior (**b**) layout of the device: 1—display; 2—housing; 3—seed chamber; 4—electronic unit; 5—light source and receiver unit.

A model of the analyzer was developed based on the schematic diagram of the device (Figure 6).

Model tests of the scarification quality control device were carried out. Dark voltage was *U*_0_ = 0.72 mV. The measurement results are presented in Table 4.

## 4. Conclusions

Mechanical scarification of forage plant seed surfaces has a significant effect on their photoluminescent properties. This may be due to damage to the near-surface layer and the opening of more luminescent seed layers, as well as a decrease in the absorption coefficient of luminescent light. At the same time, the flux increases by 1.5–1.7 times for galega, 2.0–3.0 times for clover and 2.3–3.9 times for alfalfa. Linear approximation of the flux dependence on germination with a high coefficient of determination allows us to obtain reliable linear calibration equations *B*(Φ).

The calculation of the effective return *K*_e,e_ made it possible to determine the most energy-efficient LED as a light source. Preliminary model laboratory tests allow us to talk about the effectiveness of the developed method and device, and also show the directions of its further improvement (increase in photovoltage, improvement of weight and size indicators, etc.).

The developed method and device allow not only the determination of germination during scarification, but also the increase in it by sending seeds for a repeated processing cycle if it necessary. The developed photoluminescent sensor can be used as an independent device for monitoring the germination of the scarified seeds of forage plants or as a component of a smart scarifier that determines the required number of scarification cycles. The technique needs to be validated with a larger number of samples.

## Figures and Tables

**Table 1:** Germination and photoluminescence flux of forage plant seeds with different scarifications.

Scarification	Germination *B*, %	Φ, r.u.	Φ·10^−6^, lm
Galega			
Without scarification	35	344	2.15
Single scarification	41	518	3.23
Double scarification	43	572	3.57
Clover			
Without scarification	11	505	3.15
Single scarification	15	989	6.17
Double scarification	21	1516	9.46
Alfalfa			
Without scarification	38	570	3.56
Single scarification	64	1313	8.19
Double scarification	76	2205	13.8

**Table 2:** Results of calculation of the effective output of LEDs.

LED Type [Information Source]	*K*_e,e_, %
LED 150353*BS*74500 [24]	84.4
LED *ASMT*-*AB*00-*NMP*01 [25]	83.8
LED *ASMT*-*QBB*3-*NBD*0*E* [26]	83.5
LED *KA*-3529*AQB*25*Z*4*S* [27]	82.5
LED *LZ*4-00*UA*00-00*U*6 [28]	15.8

**Table 3:** The main elements of the sensor being developed.

№	Name of the Sensor Element	Selected Item	Source of Information
1	Light source	LED 150353*BS*74500	[24]
2	Light receiver	*BPW*21*R*	[29]
3	Operational amplifier	*AD*820*ANZ*	[30]
4	Microcontroller	*ATmega328P*	[31]
5	Display	*LCD*1602	[32]

**Table 4:** The results of measuring the photon voltage *U*_ph_ when excited by light of different germination seeds after scarification.

galega			
*B*, %	35	41	43
*U*_ph_, mV	0.78	0.85	0.86
clover			
*B*, %	11	15	21
*U*_ph_, mV	0.92	1.02	1.10
alfalfa			
*B*, %	38	64	76
*U*_ph_, mV	0.85	1.08	1.12

## Data Availability

The raw data supporting the conclusions of this article will be made available by the author, without undue reservation.
